# A Fast-Response Ultraviolet Phototransistor with a PVK QDs/ZnO Nanowire Heterostructure and Its Application in Pharmaceutical Solute Detection

**DOI:** 10.3390/nano13081364

**Published:** 2023-04-14

**Authors:** Jiajun Li, Qihua Guo, Ye Tao, Dalin Li, Yiting Yang, Dandan Zhou, Jiangyong Pan, Xiang Liu, Zhi Tao

**Affiliations:** 1 School of Electronics & Information Engineering, Nanjing University of Information Science & Technology, Nanjing 210044, China; 2 College of Light Industry and Food Engineering, Nanjing Forestry University, Nanjing 210037, China

**Keywords:** phototransistor, PVK quantum dots, ZnO nanowire, pharmaceutical solute detection

## Abstract

The sensitivity and photoelectric noise of UV photodetectors are challenges that need to be overcome in pharmaceutical solute detection applications. This paper presents a new device concept for a CsPbBr3 QDs/ZnO nanowire heterojunction structure for phototransistors. The lattice match of the CsPbBr3 QDs and ZnO nanowire reduces the generation of trap centers and avoids carrier absorption by the composite center, which greatly improves the carrier mobility and high detectivity (8.13 × 10^14^ Jones). It is worth noting that by using high-efficiency PVK quantum dots as the intrinsic sensing core, the device has a high responsivity (6381 A/W) and responsivity frequency (300 Hz). Thus, a UV detection system for pharmaceutical solute detection is demonstrated, and the type of solute in the chemical solution is estimated by the waveform and the size of the output 2f signals.

## 1. Introduction

UV detection has engaged many researchers’ interest because of its wide range of applications in solar radiation monitoring and ozone layer hole and biochemical detection [1]. Similarly, in the field of medical detection, ultraviolet detection also plays an indispensable role, where ultraviolet-visible spectroscopy is generally used to detect the composition of solutions [2]. This detection method is closely related to light intensity [3]. Nevertheless, in the case of medication in children, the solute concentration of the solution used is very low. Two severe problems arise at this point: insufficient sensitivity of light intensity and interference of low-frequency noise. In order to achieve high-precision detection, pioneers invented TDLAS (tunable diode laser absorption spectroscopy), which largely overcame the low-frequency noise [4]. Current UV detectors share a common disadvantage, that is, a weak response to high-frequency signals [5]. Meanwhile, the current harmonic detection technology still has the defects of low accuracy and low signal-to-noise ratio [6,7,8].

Zinc oxide, a wide bandgap metal oxide semiconductor, has received great attention in recent years as a suitable material for photodetectors in the UV wavelength region due to the fact of its strong radiation hardness, high thermochemical stability, and wide bandgap [9,10,11]. It possesses a higher exciton binding energy (60 meV) compared to other wide bandgap materials, ensuring effective exciton emissions at ambient temperature. Furthermore, ZnO nanowires have demonstrated higher sensitivity to chemical adsorbents due to the fact of their short size comparable to its Debye length. ZnO nanostructures are now at the forefront of nanotechnology research in numerous areas [12]. The strong absorption efficiency of perovskite quantum dots over a broad spectrum range makes them ideal candidates for sensitive light detecting technology for photoelectric detection [13]. Thereby, phototransistors built on oxide semiconductors with broad bandgaps and quantum dots (QDs) with narrow bandgaps are the most efficient devices for detecting UV light photosensors [14].

This paper shares approaches to manufacturing high-performance CsPbBr3 QDs/ZnO nanowire PT (hereinafter referred to nanowire-PT) and its application in medicine solute detection systems. Compared to other ZnO-based detectors [15] (with a responsiveness of 365 A/W), a device based on PVK QDs/ZnO nanowire appears to be more efficient (6381 A/W). In addition, the perovskite QDs-based device is also inferior in front of it [16]. It turns out that the combination of ZnO nanowires and quantum dots produces surprising results due to the fact of the lattice match. This is consistent with previous results using a lattice structure and lattice match to improve a device’s performance [17,18]. Moreover, through experimental comparison, the superiority of nanowire-PT to QDs/α-ZnO PT (hereinafter referred to α-PT) is proved. Moreover, it is precisely because of the lattice adaptation that this device achieves high EQE, excellent responsivity, and outstanding detectivity. Based on this high-performance device, an integrated on-chip inspection acquisition system [19] using the detection array was created, with STM32-based ADC (analog-to-digital converter) data collection and FPGA-based multiband light source scanning. This allows for the detection of several solutes (such as penicillin, cephalosporin, and tobramycin). Therefore, this demonstration offers a straightforward, low-cost, and high-resolution approach to distinguish the UV spectra of liquid solutes.

## 2. Materials and Methods

### 2.1. Fabrication of ZnO Nanowires and CsPbBr3 QDs

The following is an illustration of the ZnO nanowire creation process: A slice of silicon (001) wafer (Zhejiang Xuchen Co. Ltd., Qu Zhou, China) was first cleaned for ten minutes in acetone, ethanol, and deionized water each. Before being moved into the chamber, the silicon wafer was placed on top of the quartz boat containing 0.2 g of zinc powder (XFnano Co. Ltd., Nanjing, China). High-quality ZnO nanowires were produced using plasmon-enhanced chemical vapor deposition (PECVD) (Jinshenweina, Beijing, China) at a regulated temperature of 800 °C, a pressure of chamber under 7.5 × 10^−3^ torr, and a flow rate of 20 sccm (Ar_2_:O_2_ = 3:1).

### 2.2. The Characterization of ZnO Nanowires and CsPbBr3 QDs

**Figure 1:**
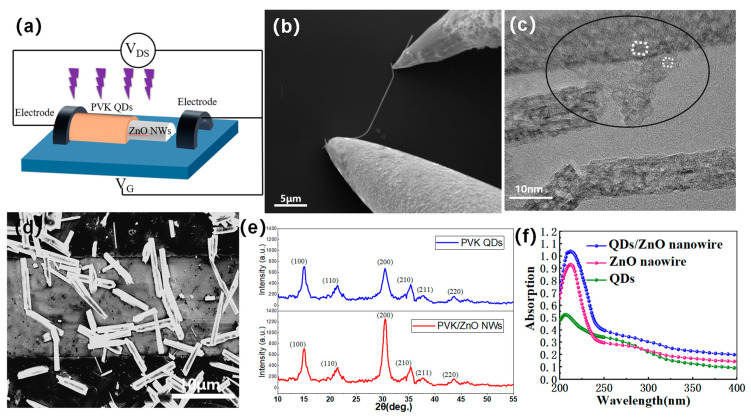
(**a**) Structure of the nanowire-PT; (**b**) SEM image of a single QDs/ZnO nanowire with PVK QDs; (**c**) TEM image of the QDs/ZnO nanowire; (**d**) SEM image of the PT’s channel; (**e**) XRD image of the PVK QDs and QDs/ZnO nanowire; (**f**) absorption spectroscopy of the QDs/ZnO nanowire, ZnO nanowire, and PVK QDs.

As seen in Figure 1b, the ZnO nanowires can be seen using SEM (Zeiss, Mainz, Germany). Then, the ZnO nanowires were mixed with octadecene, and the CsPbBr3 was fabricated by Dr. Pan [20]. The quantum dots were attached to the nanowires to obtain lattice-matched hybrid materials. The diameters of the CsPbBr3 QDs, which have clear crystal lattices and measure approximately 5 nm, are displayed in the inset of Figure 1c.

### 2.3. Manufacture of the Device

A three-terminal gated phototransistor was fabricated on the Si wafer substrate coated with 200 nm thick SiO_2_ (Zhejiang Xuchen Co. Ltd., Qu Zhou, China). Subsequently, before electroplating with the mask (W:L = 20 μm:200 μm) for the electrodes (Figure 1d), thermal annealing was performed at a temperature of 180 °C to increase the uniformity of the hybrid material layer and remove the solvent. Then, the hybrid materials were spin-coated on the substrates. For the nanowires, the uniformity was not better, and the wider diameter of the nanowires was obvious, as shown in Figure 1d. Meanwhile, as illustrated in Figure 1e, the XRD pattern (Rigaku, Japan) for the channel material, such as the PVK QDs and PVK QDs/ZnO nanowires, shows significant enhancement diffraction peaks at 31°. The broadening of the diffraction peaks is associated with grains. It can be seen from the diagram that the lattice matching of the nanowires and quantum dots had a relatively clear connection.

## 3. Results and Discussion

According to Figure 1f, the absorption intensities of the QDs/ZnO nanowire device were much higher than the ZnO nanowire device and QDs device, indicating that the combination of QDs and ZnO nanowires produces surprising effects.

**Figure 2:**
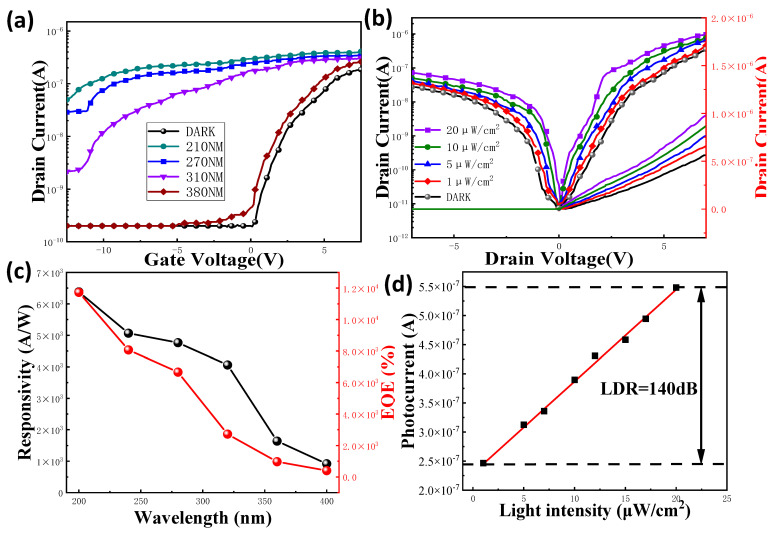
(**a**) I_D_-V_G_ graphics for the nanowire-PT at various UV wavelengths in the ultraviolet band (V_DS_ = 5 V); (**b**) at varied UV wavelengths, the density of the effective photo-induced carriers stored inside nanowire-PT; (**c**) EQE and responsivity in the UV band; (**d**) photocurrent in relation to incoming light power at wavelengths under 270 nm.

In addition, the transfer characteristics can be observed in Figure 2a, where photogenerated current (between the drain and source) is shown as a function of the gate voltage under various wavelengths of incoming light. The on/off ratio can be calculated as 10^5^. The change in the current diminishes as the incoming light moves from the low band to the high band, which is consistent with the prior absorbance characteristics of the CsPbBr3 QDs and the other functional films.

Corresponding to the above, the dimension of the channel area can be considered as 20 × 200 μm^2^. When an incident power intensity of 10 μW/cm^2^ is applied, the photoresponsivity was measured, and the transfer characteristic curve is provided in Figure 2a. This photoresponsivity may be computed using Equation (1) [21].

(1) $$R=\frac{I_{total}-I_{dark}}{P}=\frac{I_{ph}}{\rho \cdot S}$$

where P represents the optical power, I_dark_ is the dark current, I_total_ denotes the total current, ρ indicates the optical incident power density, I_ph_ implies the photocurrent, and S is the effective area for the photo-electric reaction. By calculating the responsivity of the different wavelengths, as shown in Figure 2c, with the deviation of the incidence wavelength in the UV band, the responsiveness fell from 6381 A/W to 534 A/W. The EQE (external quantum efficiency) decreased from 11,734% to 256% at the same moment.

(2) $$\mathrm{EQE}=\frac{I_{ph}/q}{\rho /h\nu }\times 100\%$$

Upon attempting to explain the current phenomenon of photosensitivity of the TFT at UV wavelengths, it is necessary to examine the behavior of the response as a function of the optical power, which may indicate the prospective applications of excellent sensors, as well as the characterization of the PT sensitivity. In a logarithm/logarithm plot, Figure 2b,d show the fluctuation of the photocurrent for a 270 nm wavelength as a function of the light power. Furthermore, based on the light intensity-dependent photoresponse properties (Equation (3)) [22], the device’s liner dynamic range (LDR) is estimated to be 140 dB.

(3) LDR=20log(IphIdark)

LDR denotes the device’s operational range for light intensity. Hence, a comparatively large LDR denotes a device with minimal recombination losses [23].

The photodetector’s response frequency is related to the photogenerated carrier transfer efficiency, which could be influenced by the noise. The charge carrier mobility fluctuations, generally known as flicker noise, as they are induced by the capture and release of carriers, are the main cause of noise in semiconductors [24]. In heterostructure photodetectors, the traps are located at the interface between materials. Concurrently, lattice mismatch will lead to the degradation of long-term reliability and the degradation of trap density, so the performance of lattice matches must be the best as possible for hybrid materials. The evaluation the lattice matches can be calculated by the electric performance of the photodetectors.

**Figure 3:**
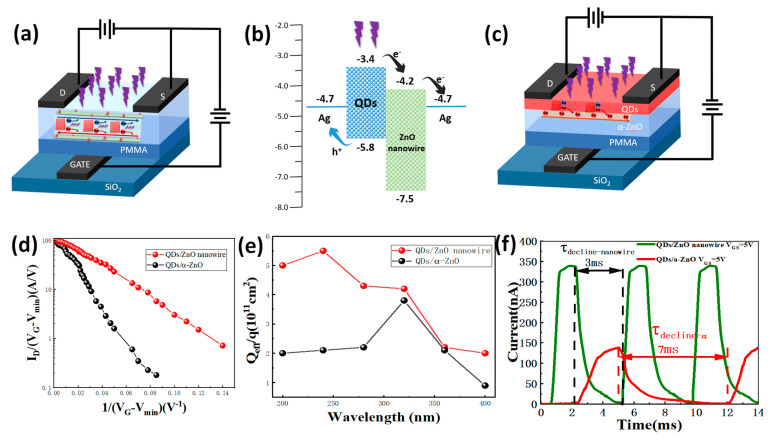
(**a**,**c**) Comparison chart of the QDs/ZnO nanowire PT and QDs/α-ZnO PT (hereinafter referred to as nanowire-PT and α-PT); (**b**) band diagram and charge transfer diagram of the hybrid material; (**d**) semilogarithmic plot of I_D_/(V_G_ − V_min_) versus 1/(V_G_ − V_min_) for the phototransistor with or without incident light (270 nm, peak power = 5 μW/cm^2^); (**e**) efficient light-induced carrier density stored in optical transistors at various UV wavelengths; (**f**) comparison of the nanowire-PT and α-PT’s light signal responses at 270 nm and 5 μW/cm^2^ under a 5 V gate voltage.

For Figure 3a, due to the lattice matching of the CsPbBr3 QDs and ZnO nanowire, the hanging key on the interface is avoided as a trap center, and the composite center hardly absorbs carriers, which greatly improves the injection efficiency and quantum efficiency. In addition, this increases the carrier mobility and improves the device’s performance. Compared with the QDs/α-ZnO device (Figure 3c), the lattice structure of the nanowire-PT is more compact. As a result, the photo current of the nanowire-PT is more obvious (Figure 3d,e).

The energy band schematics of the device are presented in Figure 3b. The charge transfer takes place across the interfaces of the CsPbBr3 QDs/ZnO nanowires. CsPbBr3 QDs are thought to have an optical property that allows carriers to be created and transported from the CsPbBr3 QDs to the ZnO nanowires in the CsPbBr3 QDs/ZnO nanowire hybrid materials. The charge transferred into the ZnO nanowire (ECB = −4.2 eV, EVB = −7.5 eV) and then drifted to the source electrode under the bias before the electron–hole pairs in the CsPbBr3 QDs recombine. The drain electrode can supply an equivalent number of carriers in the interim to support the channel’s conversation of charge. As the vacuum energy level is regarded as the standard for potential energy, the valence band (EVB) and conduction band (ECB) of the CsPbBr3 QDs, which were −4.1 and −6.2 eV, could also be measured [25]. Heterojunction barriers in nanowire-PT can lessen the photoelectric noise while simultaneously increasing the efficiency of opto-electric conversion.

As illustrated in Figure 3d, the semilogarithmic plot for the CsPbBr3 QDs/ZnO nanowire with illumination transformation could be applied to compute defect the density for the following step. There is a linear range in both curves. The linear functions’ slope is smaller for illumination than for dark current, noting a lower slope for less defect density, and the deviation from linearity occurs at greater 1/(V_G_ − V_min_) [26].

(4) $$I_{D}=\frac{W}{L}\mu_{0}C_{ins}\left(V_{G}-VD\exp\left(\frac{q^{3}{N_{T}}^{2}t}{8\varepsilon_{0}\varepsilon_{sc}KTC_{ins}(V_{G}-{V_{min}}_{min})}\right)\right)$$

The channel’s width and length are W and L, respectively. The gate insulator’s capacitance per unit of area is known as C_ins_. V_min_ is the gate voltage at the lowest possible I_D_, where the channel first forms before I_D_ gradually increases. The drain voltage is V_D_. The electronic charge is q. The channel’s thickness is t. The surface accumulation of flaws in the grain boundaries is known as N_T_. S is the semiconducting active layer’s permittivity. The thermal energy unit Is K_T_. A linear function might be employed to fit the semilogarithmic curve of I_D_/(V_G_ − V_min_) versus 1/(V_G_ − V_min_), derived using Equation (4). The function’s slope varies proportionally to N_T_^2^.

In addition, photoexcited effective charge densities are revealed by investigating the stored efficient charges [27].

(5) $$\Delta Q_{eff}(\varepsilon )=C_{ox}\cdot \Delta V_{th}(\varepsilon )$$

Compared to α-PT, the nanowire-PT devices exhibited stronger optical carrier memory capacity at various wavelengths. The plots in Figure 3e show that while a small ΔV_th_ was seen below 275 nm, the nanowire-trap PT’s density was minimal.

To summarize, due to the lattice match of the CsPbBr3 QDs and ZnO nanowire, the structure of the interface transition zone improved excellently, which also increased the carrier mobility. In addition, the great repeatability and rapid photoresponse in dynamic incident light detection are significant benefits for the integrated PT’s practical application. Figure 3f depicts the dynamic photocurrent characteristics of the studied devices with varied gate voltages. In a previous report [28], for QDs/α-ZnO, there was an absorption peak at 100 Hz. However, nanowire- PT still has a peak in absorption at 300 Hz. So, it was tested at a frequency environment of 300 Hz. The response and decline times of the nanowire-PT were estimated to be 1 and 3 ms, respectively. The photocurrent response time was affected by the optical carrier injection mechanism. In comparison to α-PT’s decline time (7 ms), the nanowire-PT’s decline time (3 ms) was shorter due to the lattice match.

**Figure 4:**
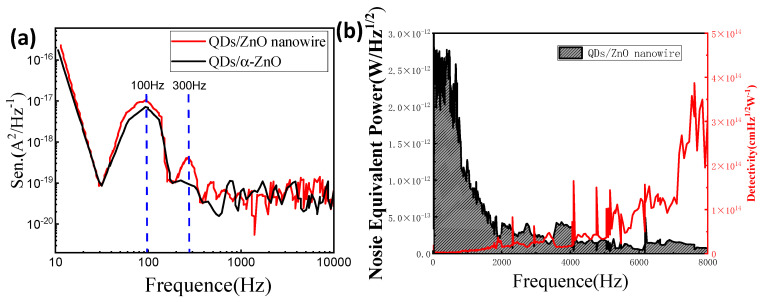
(**a**,**b**) The improved nanowire-PT’s noise equivalent power (NEP) and detectivity spectrum measured in relation to the gate frequency at 300 Hz under a dynamic detecting environment.

To determine the detectivity of the current devices for comparison, the detectivity D* is the sensor’s capacity to detect a weak signal, especially under dynamic detecting conditions. It describes the sensor in relation to its noise, as defined by the noise current spectrum shown above. The following determines the detectivity [29]:

(6) $$NEP=\frac{I_{N}}{R}(wHz^{-\frac{1}{2}})$$

(7) $$D^{*}=\frac{\sqrt{A}}{NEP}(cmHz^{\frac{1}{2}}/w,Jones)$$

where NEP means the noise equivalent power, I_N_ implies the noise current density, A represents the active area, and R denotes the responsiveness. As shown in Figure 4a, due to the tight lattice structure in the heterostructure and the adaptation of nanowires to quantum dots, the detection rate of the nanowire-PT was at the lowest noise density level. The final dynamic performances of the nanowire-PT can be calculated and are displayed in Figure 4b.

(8) $$\int_{0}^{\infty }NEP(f)d(f)=NEP(f)\Delta (f)$$

The modulation bandwidth was 300 Hz, as determined by the experiment phenomena, demonstrating the impact of the touched interface inside the channel. Under a dynamic detecting environment, the detectivity of the nanowire-PT concurrently reached 8.13 × 10^14^ Jones.

Our nanowire-PT obtained exceptional responsiveness and photodetectivity in comparison to the published UV phototransistor listed in Table 1. This nanowire-PT is additionally suitable for solute detection.

**Figure 5:**
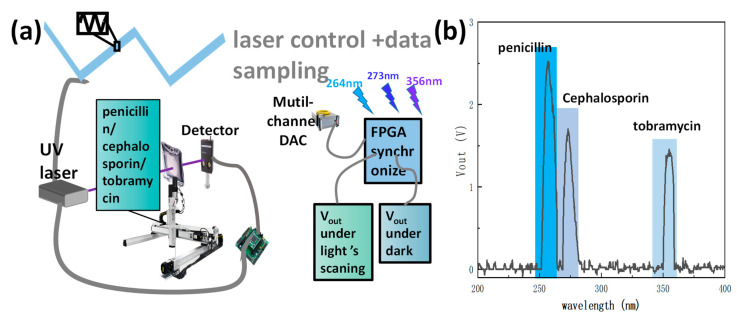
(**a**) Schematic and contrast images of the solute sensing systems; (**b**) real-time multiplexed sensing of a solute mixture consisting of 500 μg/mL penicillin, 500 μg/mL cephalosporin, and 500 μg/mL tobramycin.

For potential applications in UV band light detection, Figure 5a shows the device for solute detection. Owing to the DFB laser’s highly narrow monochromaticity [33], the system is enabled to realize the recognition of certain solutes and can also be applied to ultraviolet spectroscopy detection. Varying with the operating current, FPGA allows for the synchronization of the UV laser’s wavelength to the optical inverter’s sampling frequency. In the ultraviolet range of the absorption spectra of distinct solutes, the response band of the collected optical inverter demonstrated a substantial overlap.

Such solutes include penicillin, cephalosporin, and tobramycin. Therefore, multi-wavelength UV lasers and ultraviolet light inverters were assembled into a TDLAS (tunable diode laser absorption spectroscopy) approach for solute detection [34]. This phototransistor can serve as a component of high-frequency WMS (wavelength modulation spectroscopy) solute sensing technology owing to the phototransistor’s rapid reaction time (Figure 3f) [35]. The modified Beer–Lamber law equation can be utilized to determine the function between the detecting signal and the solute concentration [36,37]. FA = V_GO_/V_sat_. Therefore, FA is the absorbance fraction of the detected solute; V_GO_ is the response voltage offset of the phototransistor. When the concentrations are extremely high, V_sat_ is the saturated D voltage. By distinguishing the solute composition of the mixed liquid (500 μg/mL penicillin, 500 μg/mL cephalosporin, and 500 μg/mL tobramycin), the phototransistor is measured under the adjacent wavelength modulation of the UV laser (Figure 5b).

**Figure 6:**
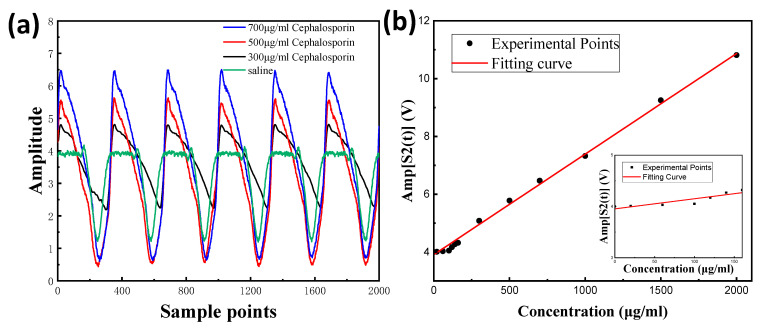
(**a**) Various cephalosporin concentrations represented by distinct 2f signals in systems with nanowire-PT; (**b**) 2f signal amplitude as a function of cephalosporin concentration.

The tremendous 2f signals can be observed In Figure 6a, with various cephalosporin concentrations, which is consistent with the image data in Figure 5b. The amplitude of the 2f signal and cephalosporin concentration can be matched roughly linearly, (A = 3.91227C + 0.00348 (unit: V)), as observed in Figure 6b. At very low concentrations, the amplitude of the 2f signal and cephalosporin concertation can be modified with (A = 3.95276C + 0.04244 (unit: V)) for the modified signal and solvent influence [38]. Measured experimentally, the lowest detection limit (LOD) of cephalosporin can ascend to 140 μg/mL, which suggests that our device can implement the precise differentiation of the solute species in mixed liquids.

## 4. Conclusions

In summary, a CsPbBr3 QDs/ZnO nanowire-based, high-precision, and low-optoelectronic noise phototransistor with 6381 A/W responsivity was successfully developed. The device’s reaction time may be lowered to 1 ms at 5 V, while the decline time was 3 ms. Its maximal specific detectivity was predicted to be 8.13 × 10^14^ Jones under 5 V at 270 nm, and the EQE could reach up to 11,734%. Thanks to the lattice matching, the device attained a significant LDR (140 dB). In addition, this finding opens the door for the utilization of nanowire-PTs with simple structures, high accuracy, and excellent detectivity. It can work at relatively low concentrations (140 μg/mL). Finally, the device was applied to detect solutes in solution and select several solutes to measure the specific output of 2f waveforms.

## Figures and Tables

**Table 1:** Contrasting this research with other phototransistors described in the literature.

Materials	Response Time (ms)	Responsivity (A/W)	Detectivity (Jones)	Reference
CsPbBr3 QDs/ZnO nanowire	1	6381	8.13 × 10^14^	This work
PEDOT:PSS/SnO_x_/IGZO	<500	984	3.3 × 10^14^	[30]
a-Ga_2_O_3_	-	4100	2.5 × 10^13^	[31]
ZnO/SnO_2_	2070	82.28	7.79 × 10^13^	[32]

## Data Availability

Not applicable.
